# Hypnal or Monochloral-Antipyrin

**Published:** 1893-02-25

**Authors:** 


### NOTES ON NEW DRUGS.

### HYPNAL OR MONOCHLORAL-ANTIPYRIN.

Many new and valuable hypnotic drugs have been introduced within the last few years, and while in many we find certain shortcomings and contraindications, we have made great additions to our therapeutical resources. We have found hypnal very useful in several cases where we have been anxious to avoid the dangers of chloral, or in cases in which an analgesic was required to overcome pain which would have probably rendered the ordinary doses of chloral inert.

Hypnal is, however, but little known, so much so that on several occasions we have had considerable difficulty in getting it dispensed without delay in such a large city as Bristol, for the simple reason that many chemists have never been asked for it. We therefore hope that some brief references to the pharmacology and clinical experiences of hypnal will prove of service to our readers.

The *Ph. J.*, 1890, 889, says that recently Herr Reuter showed that by heating antipyrin with chloral hydrate a crystalline compound could be obtained, which he described as trichloraldehyd phenyldimethyl pyrazol, and alleged to be without therapeutic value. According, however, to the experience of Dr. Bardet, recently communicated to the Société de Thérapeu ticque (*Nouv. Rem.*, March 24th, p. 135), this crystalline compound, which he proposes to call "hypnal," partakes in a marked degree of the properties of both its constituents.

Administered in twenty-two cases in doses of one gram (two grams being rarely required) he found hypnal to induce sleep as readily as chloral hydrate, whilst in those instances where the insomnia was caused by pain it seemed to have the same anodyne effect as antipyrin.

In addition, spasmodic symptoms, especially cough, appeared to be much abated under its influence.

Dr. Bardet states that hypnal consists of about 45 per cent. of chloral and 55 per cent. of antipyrin. It has been found by M. Bonnet that if concentrated solutions of the two constituents be shaken together a considerable deposit of crystals is formed without passing through the oily stage, and this deposit, by re-crystallisation from water, can be obtained in enormous transparent rhombic crystals.

If the chloral used be in excess the crystals take the form of prismatic needles. The compound is said to be free from odour or caustic taste. In fact, according to M. Bonnet, it is tasteless; but Dr. Bardet says that while it is free from the strong taste of chloral hydrate and the bitterness of antipyrin, it has a saline taste, a slight but not disagreeable sensation of chloral becoming perceptible on the tongue after some time. Hypnal is said to dissolve in six to eight times its weight of warm water, and melt at 58°—60°. It is, therefore, much less soluble than chloral hydrate or antipyrin, while as to its melting point, it lies about half-way between the two. When heated with a dilute base, chloral and antipyrin are reformed, and upon heating the solution the characteristic odour of chloroform is given off. It may be added, that since the appearance of the last note in *The Month* (February 1st, p. 602) upon the subject, Herr Reuter has represented the combination that takes place by the following equation, in which the chloral is made to displace in one of the methyl groups of antipyrin an atom of hydrogen, which, with an hydroxyl of the chloral hydrate, forms water:

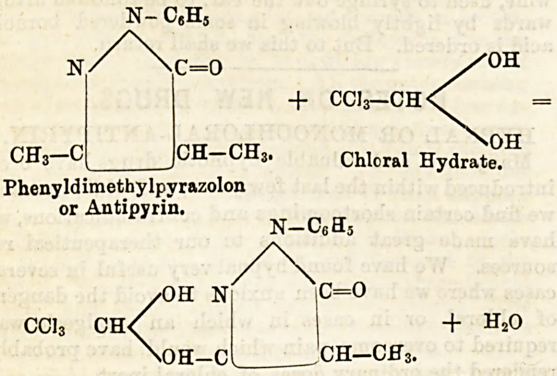

Trichloraldehyd-phenyldimethyl pyrazolon or Hypnal.

In *The Month*, May 31st, 1890, page 977, a reclamation for priority has been made by Messrs. Béhal et Choay, on account of the discovery of the compounds formed by antipyrin with chloral hydrate (*Journ. Pharm. Chim.*, May 15th, p. 539). They point out that in the Paris Exhibition of last year two such compounds were exhibited, and they state that the compound described by Herr Reuter is a third modification that no longer gives the reactions of antipyrin. As has been before mentioned, when concentrated solutions of equal weights of chloral hydrate and antipyrin are mixed, an oily layer is formed which, after a short time, crystallises. If the quantities used be in the proportion of 4·7 grams of chloral hydrate and 5·3 grams of antipyrin, dissolved each in 5 grams of water by the aid of a gentle heat, large crystals may be obtained of a compound containing equal molecules of antipyrin and chloral, and represented when crystallised from alcohol by the formula $C_{13} H_{13} N_2 Cl_3 O_2$.

This has been designated "monochloralantipyrin." It melts at 67°—68° C., dissolves in water at 14° C. to the extent of 7·85 grams in 100 grams, gives with ferric chloride the characteristic blood-red colour of antipyrin, gives rise to chloroform when heated with potash in aqueous solution, and reduces Fehling's solution when warmed. If this compound be kept heated to about its fusing point for a time there are gradually deposited in the melted mass crystals of a compound corresponding to a product of dehydration, and this, according to Messrs. Béhal and Choay, is the substance obtained by Reuter. It is insoluble in water, melts at 186°—187° C., and can be distinguished easily from monochloral-antipyrin by no longer giving the reaction with ferric chloride.

If, instead of using the constituent compounds in equal molecular proportions a concentrated solution of chloral hydrate be used in excess, an oily layer is similarly formed, from which prismatic needles are obtained containing two molecules of chloral to one of antipyrin, and therefore named "bichloral antipyrin." This also melts at 67°—68°, but is more soluble than monochloral antipyrin, the proportions being 9·98 grams in 100 grams of water at 14° C. It gives with ferric chloride the red colouration of antipyrin, and reduces Fehling's solution when heated. In water it appears to undergo some degree of dissociation, the first crystals deposited from a saturated solution being those of monochloral antipyrin, and afterwards those of the bichloral compound.

